# Potent Antifungal Functions of a Living Modified Organism Protein, CP4-EPSPS, against Pathogenic Fungal Cells

**DOI:** 10.3390/molecules28114289

**Published:** 2023-05-24

**Authors:** Seong-Cheol Park, Hye Song Lim, Seong-Eun Mun, Young Jun Jung, A-Mi Yoon, Hyosuk Son, Chul Min Kim, Young-Kug Choo, Jung Ro Lee

**Affiliations:** 1 Department of Chemical Engineering, Sunchon National University, Suncheon 38286, Republic of Korea; schpark9@gnu.ac.kr; 2 LMO Team, National Institute of Ecology (NIE), Seocheon 33657, Republic of Korea; hslim0826@nie.re.kr (H.S.L.); june5763@nie.re.kr (Y.J.J.); pinus457@nie.re.kr (A.-M.Y.); 3 Department of Horticulture Industry, Wonkwang University, Iksan 54538, Republic of Korea; chulmin21@wku.ac.kr; 4 Department of Biological Science, College of Natural Science, Wonkwang University, Iksan 54538, Republic of Korea; showmse@naver.com (S.-E.M.); ykchoo@wku.ac.kr (Y.-K.C.); 5 Division of Life Sciences, Jeonbuk National University, Jeonju 54896, Republic of Korea; 6 Department of Exhibition and Education, National Marine Biodiversity Institute of Korea, Seocheon 33662, Republic of Korea; skitten74@mabik.re.kr

**Keywords:** CP4-EPSPS, antifungal protein, *Agrobacterium*, living modified organism

## Abstract

Various proteins introduced into living modified organism (LMO) crops function in plant defense mechanisms against target insect pests or herbicides. This study analyzed the antifungal effects of an introduced LMO protein, 5-enolpyruvylshikimate-3-phosphate synthase (EPSPS) from *Agrobacterium* sp. strain CP4 (CP4-EPSPS). Pure recombinant CP4-EPSPS protein, expressed in *Escherichia coli*, inhibited the growth of human and plant fungal pathogens (*Candida albicans*, *C. tropicalis*, *C. krusei*, *Colletotrichum gloeosporioides*, *Fusarium solani*, *F. graminearum*, and *Trichoderma virens*), at minimum inhibitory concentrations (MICs) that ranged from 62.5 to 250 µg/mL. It inhibited fungal spore germination as well as cell proliferation on *C. gloeosporioides*. Rhodamine-labeled CP4-EPSPS accumulated on the fungal cell wall and within intracellular cytosol. In addition, the protein induced uptake of SYTOX Green into cells, but not into intracellular mitochondrial reactive oxygen species (ROS), indicating that its antifungal action was due to inducing the permeability of the fungal cell wall. Its antifungal action showed cell surface damage, as observed from fungal cell morphology. This study provided information on the effects of the LMO protein, EPSPS, on fungal growth.

## 1. Introduction

Living modified organisms (LMOs), commonly called genetically modified organisms (GMOs), are widely used in agriculture to enhance crop quality and yield by introducing new traits, such as insect resistance, herbicide tolerance, increased yield, and resistance to abiotic (cold, heat, and soil degradation) and biotic stressors (diseases and insect pests) [1,2,3,4,5]. Although most LMO crops have been designed to defend against target insect pests or herbicides, the potential unexpected effects of LMOs on the natural ecosystem or human health remains unclear [6], particularly in the case of unintended release into the natural ecosystem, which may alter biodiversity through food chain transfer, gene flow, gene transfer, and root exudates [7,8,9,10,11].

One of the benefits of using 5-enolpyruvylshikimate-3-phosphate synthase (EPSPS, EC2.5.1.19) or phosphinothricin-acetyltransferase (PAT) in LMO crops is the ability to grow the plants while using chemical herbicides. Glyphosate, the active ingredient in Roundup, is a broad-spectrum herbicide that inhibits EPSPS, an enzyme responsible for catalyzing the penultimate step in the shikimate pathway, which synthesizes the three aromatic amino acids (phenylalanine, tyrosine, and tryptophan) in most prokaryotes, plants, and fungi. Roundup Ready crops (Monsanto Company, St. Louis, MO, USA) contain the *CP4-EPSPS* gene, which encodes a glyphosate-tolerant enzyme originally found in *Agrobacterium* sp. strain CP4; EPSPS transfers the enolpyruvyl moiety of phosphoenolpyruvate (PEP) to the 5-hydroxyl group of shikimate-3-phosphate (S3P) [12,13,14]. Herbicide-resistant crops are cultivated in more than 94% of the world’s transgenic crops annually. Among them, glyphosate-resistant canola, cotton, soybean, maize, and sugar beet crops are major agricultural products. CP4-EPSPS enzyme has both intrinsic tolerance to glyphosate and a high affinity for PEP. 

The potential impact of the unintended release of herbicide-resistant LMO crops containing EPSPS or PAT proteins on the natural populations of soil microorganisms, including fungi and bacteria, raises concerns [15,16]. The root exudates and mucilage of LMO crops may affect soil microorganisms [17,18], making it crucial to comprehensively understand the functions of LMO proteins, like EPSPS, to minimize the potential effects of approved LMOs on the natural environment. On the other hand, if the EPSPS protein has other functions, such as antimicrobial activity, we may suggest its use in the possibile development of pathogen-resistant LMO crops. In particular, fungi attack several important crops, causing damage and reducing their productivity. Besides herbicides, treating other chemical drugs to combat fungi on crops may also lead to soil environmental degradation. However, there are no commercial transgenic plants with genes encoding proteins to confer resistance against fungi though several studies have already described the efficacy of antimicrobial peptides (AMPs) in transgenic plants [19,20,21,22].

AMPs are critical in the innate host defense mechanism of most organisms as a primary barrier against pathogenic infections [23,24,25]. As well as antibacterial peptides, hundreds of antifungal peptides and proteins are known, divided into several classes of antifungal proteins [26,27,28,29,30]: (1,3)β-glucanases, chitinases, cyclophilin-like proteins, defensins, killer proteins (killer toxins), lipid-transfer proteins (LTPs), PR-1 proteins, protease inhibitors, ribosome-inactivating proteins, thaumatin-like (TL) proteins, and the others. Most antifungal proteins act on the fungal cell wall. The antifungal activity of plant (1,3)β-glucanases occurs by hydrolyzing the (1,3)β-glucan present in the fungal cell wall, resulting in cell lysis and cell death [27]. Chitinases that cleave cell wall chitin polymers in situ can render fungal cells osmotically sensitive [31]. TL proteins are fungicidal against a broad spectrum of plant and human pathogens and cause cell permeability changes in fungal cells with a cell wall [27,32]. Fungal defensins are involved in fungal inhibition that occurs by ion efflux via binding to specific cell membrane receptors [33]. The mechanisms of other antifungal proteins are not fully understood. 

Most AMPs have amphipathic structures with hydrophilic and hydrophobic interfaces to combat pathogenic microorganisms. Furthermore, recent research has shown that secondary structures of EPSPS proteins exhibit characteristics similar to the overall features of AMPs, in that they contain an α-helix, β-sheet, and flexible loop [12]. This study aimed to confirm the antimicrobial activity of the CP4-EPSPS protein and determine its potential use as an antibiotic. By analyzing the antimicrobial effect of the CP4-EPSPS protein on various fungi, this study contributes to developing sustainable and effective biological control strategies in agriculture.

## 2. Results and Discussion

### 2.1. Expression and Purification of Agrobacterium EPSPS Protein

**Figure 1:**
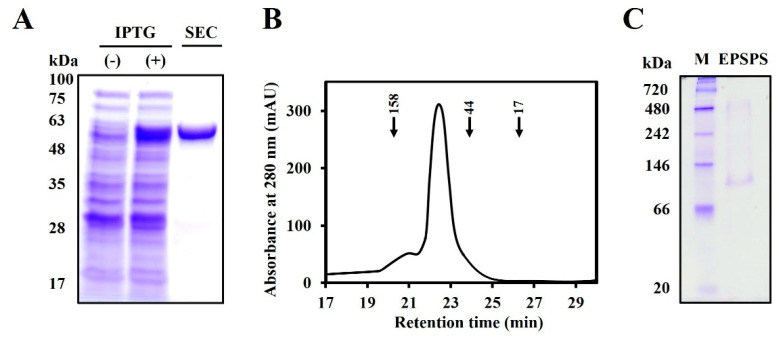
Characterization and purification of 5-enolpyruvylshikimate-3-phosphate synthase (EPSPS) from *Agrobacterium* sp. strain CP4 (CP4-EPSPS) protein. The recombinant CP4-EPSPS protein expressed by *E. coli* was analyzed using 12% sodium dodecyl sulfate-polyacrylamide gel electrophoresis (SDS-PAGE) (**A**) stained with Coomassie blue. SEC: pure protein fractionated from size exclusion chromatography (SEC) (**B**) Purification and structural analysis of the CP4-EPSPS protein using SEC after affinity chromatography. SEC was performed using fast protein liquid chromatography (FPLC) on an Enrich SEC 650 column. Arrow: protein standard. (**C**) The purified CP4-EPSPS protein (10 μg) was analyzed on native-PAGE. M: standard marker.

During the growth process of unintentionally released genetically modified plants into the natural environment, they are exposed to various soil microorganisms and may secrete LMO proteins such as Crystal (Cry) and EPSPS [15,34,35]. This study aimed to determine the physiological functions of CP4-EPSPS, a well-known LMO protein derived from *Agrobacterium* sp. CP4 line. The full-length CP4-EPSPS was expressed in *E. coli* BL21 (DE3) by adding IPTG, and the CP4-EPSPS recombinant protein was purified using Ni-NTA affinity and size exclusion chromatography (SEC). SDS-PAGE analysis revealed the effectiveness of this purification step (Figure 1A). The oligomeric structure of the CP4-EPSPS protein was observed using SEC (Figure 1B) and native-polyacrylamide gel electrophoresis (native-PAGE) (Figure 1C) analysis, which revealed that the CP4-EPSPS protein formed dimeric complexes. These results mean that the isolated CP4-EPSPS protein was pure. 

### 2.2. Antifungal Activity of CP4-EPSPS

A microtiter plate assay was conducted to evaluate the antifungal activity of the purified recombinant CP4-EPSPS protein and melittin with human fungal pathogens (*C. albicans*, *C. tropicalis*, and *C. krusei*) and plant fungal pathogens (*C. gloeosporioides, F. solani*, *F. graminearum*, and *T. virens)* and their minimum inhibitory concentrations (MICs) were determined. The CP4-EPSPS protein inhibited the growth of the tested yeast and mold fungi with MIC values ranging from 62.5 to 250 μg/mL (Table 1); this antifungal activity was superior to that of melittin, the antifungal peptide used as a control. The antifungal activity of CP4-EPSPS was better in mold cells than in yeast cells, except *C. krusei*. This suggests that differences in antifungal capacity may appear depending on the components of the cell wall of fungi.

**Figure 2:**
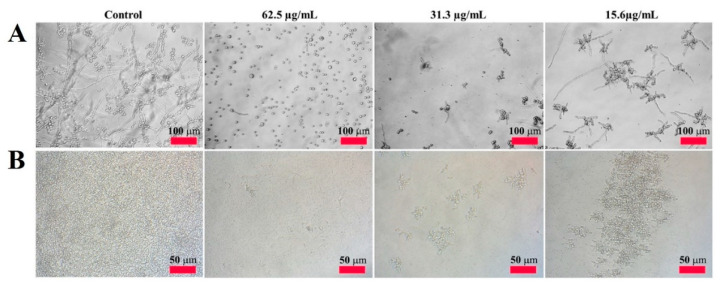
Antifungal activity of the CP4-EPSPS protein against *C. gloeosporioides* (**A**) and *C. krusei* (**B**). Concentration-dependent growth inhibition of CP4-EPSPS in liquid cultivation was visualized under a microscope. Bar is 50 µM.

To visualize the antifungal activity of CP4-EPSPS, we monitored the hyphal growth of the mold fungi and proliferation of the yeast in liquid culture. As shown in Figure 2A-control, *C. gloeosporioides* without protein had spore germination and were growing in multiple branches for 24 h. In *C. gloeosporioides* treated with CP4-EPSPS, spore germination was completely inhibited at 62.5 µg/mL, and the spore size was smaller than that of the control, indicating direct cell damage. CP4-EPSPS inhibited spore germination in a concentration-dependent manner, even at low concentrations. Figure 2B shows its inhibitory action on the proliferation of *C. krusei*. It also completely inhibited the proliferation of *C. krusei* at 62.5 µg/mL. 

**Figure 3:**
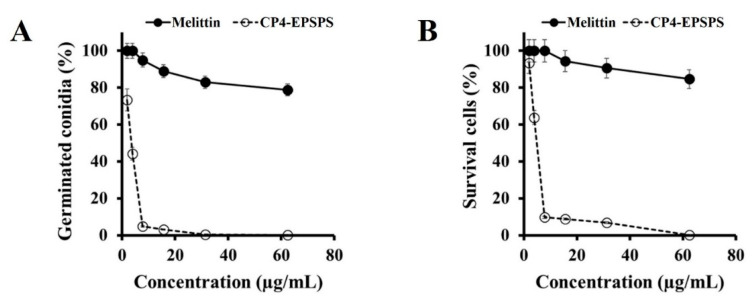
Dose-dependent effects of the CP4-EPSPS protein and melittin on conidia germination and survival of *C. gloeosporioides* conidia. (**A**) Germinated *C. gloeosporioides* conidia were quantified using microscopy after 24 h of sample incubation. (**B**) After 24 h of sample treatment, survival cells of *C. gloeosporioides* were counted using flow cytometry using propidium iodide (PI) dye.

To determine whether CP4-EPSPS affects the survival or conidia germination of *C. gloeosporioides* cells, we evaluated the germination and survival percentages of conidia cells by counting non-germinated conidia on a hemocytometer under a microscope and uptake of PI dye using flow cytometry. The CP4-EPSPS protein significantly suppressed conidial germination and survival of the conidia of *C. gloeosporioides* in a dose-dependent manner, compared to melittin (Figure 3A,B). This result indicates that the CP4-EPSPS protein directly acts on *C. gloeosporioides* cells and is a fungicidal protein.

### 2.3. Subcellular Localization of CP4-EPSPS in Fungal Cells

**Figure 4:**
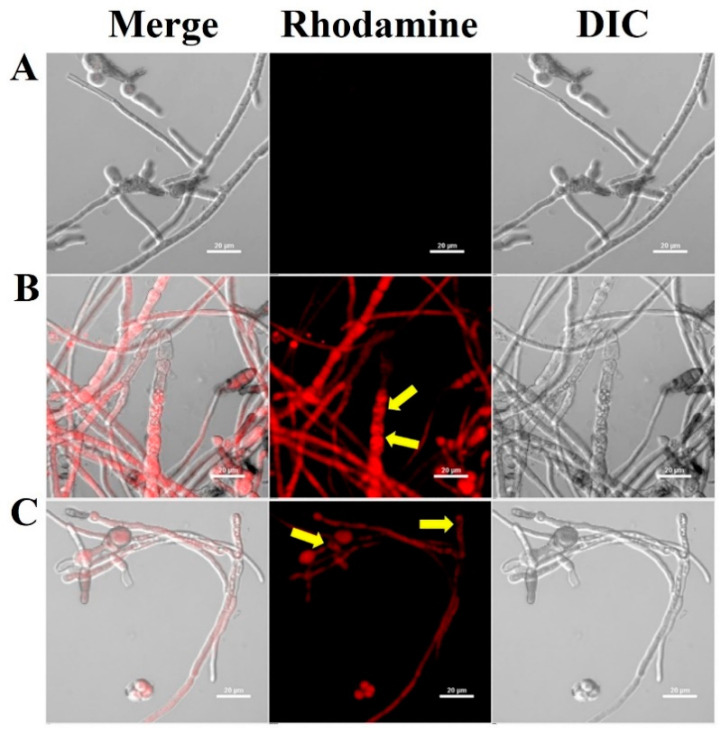
Localization of rhodamine-labeled CP4-EPSPS protein in *C. gloeosporioides* hyphal cells. Representative confocal images showing cells without protein (**A**) and with rhodamine-labeled CP4-EPSPS (**B**) and rhodamine-labeled melittin (**C**) of minimum inhibitory concentrations (MICs) in conidial germlines. Intracellular accumulation of rhodamine-labeled proteins was observed under a confocal laser scanning microscope. Yellow arrows indicate the damaged cell surfaces.

The initial binding affinity of AMPs to fungal cells is a critical factor in determining their antifungal effect [36,37,38]. To investigate the effect of the antifungal effect of CP4-EPSPS protein on the cell wall and membrane or translocation into the cytoplasm, the cellular distributions of the rhodamine-labeled CP4-EPSPS in *C. gloeosporioides* were observed using CLSM (Figure 4). Rhodamine-labeled CP4-EPSPS caused cell surface and cytosolic fluorescence accumulation in spores and hyphae in the treated cells, demonstrating that the CP4-EPSPS protein was translocated across the cell membrane and into the cytosolic space.

### 2.4. Cellular Uptake of CP4-EPSPS Protein via Membrane Integrity Change

**Figure 5:**
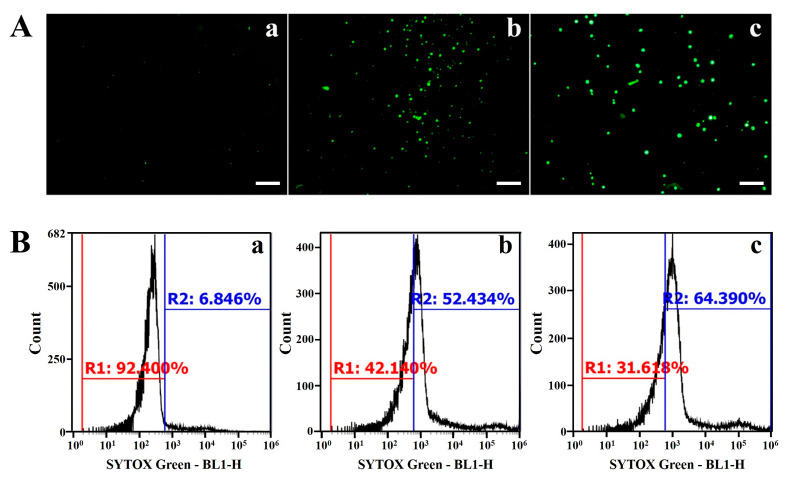
Membrane permeability of CP4-EPSPS and melittin in *C. gloeosporioides* cells. (**A**,**B**) After treatment with phosphate-buffered saline (PBS) (**a**), CP4-EPSPS (**b**), or melittin (**c**) at MIC for 2 h, SYTOX Green was added to the cells (0.5 µM); the cells were monitored under a fluorescence microscope (**A**) and evaluated using flow cytometry (**B**). Bar is 50 µM.

The cytosolic localization of AMPs through the cell wall and membrane can occur through direct penetration, partial plasma membrane disruption, vacuolar localization, transition pore formation, and endocytosis [39]. To investigate whether the CP4-EPSPS protein was translocated into the cytosol through membrane-damaging or non-damaging activity, a membrane-impermeable probe, SYTOX Green, which fluoresces green when attached to nucleic acids, was used. This dye is able to enter the cells when the cell membrane integrity is compromised or when holes are formed by antifungal agents [40]. Flow cytometry and fluorescence microscopy were used to assess the effect of the CP4-EPSPS protein on membrane integrity. After incubating *C. gloeosporioides* cells with CP4-EPSPS for 2 h, intracellular green fluorescence was observed in over 50% of the cells (Figure 5A), indicating the membrane permeability of CP4-EPSPS and melittin in *C. gloeosporioides* cells (Figure 5B). These results suggest that membrane integrity disruption allows the CP4-EPSPS protein to pass through the cell wall and accumulate in the cytoplasm of fungal cells.

**Figure 6:**
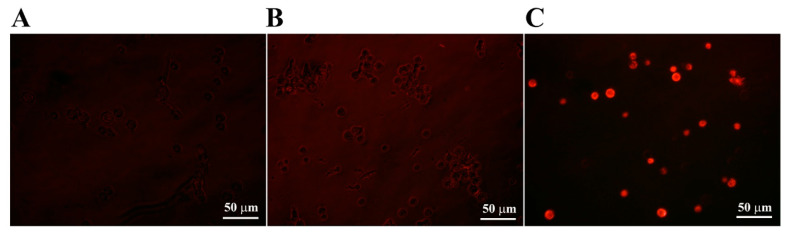
Mitochondrial reactive oxygen species (ROS) generation in *C. gloeosporioides* cells. After 4 h incubation with PBS (**A**), CP4-EPSPS (**B**), or melittin (**C**), the MitoSOX probe was added to the cells (5 µM), and the cells were observed under a fluorescence microscope.

Mitochondria are crucial in controlling cell death and inordinate ROS generation; they can induce the opening of the permeable transition pore, cytochrome c release, and caspase system activation, leading to apoptosis [37]. To investigate the intracellular events induced by the CP4-EPSPS in fungal cells, ROS generation in the mitochondria of CP4-EPSPS-treated *C. gloeosporioides* cells was assessed using MitoSOX Red, a mitochondrial superoxide indicator (Figure 6). The microscopic fluorescence images revealed a considerable increase in MitoSOX Red fluorescence intensity in melittin-treated cells but not in CP4-EPSPS-treated cells. This result indicated that the CP4-EPSPS-induced fungal cell death might be due to direct membrane breakage rather than mitochondrial ROS generation.

**Figure 7:**
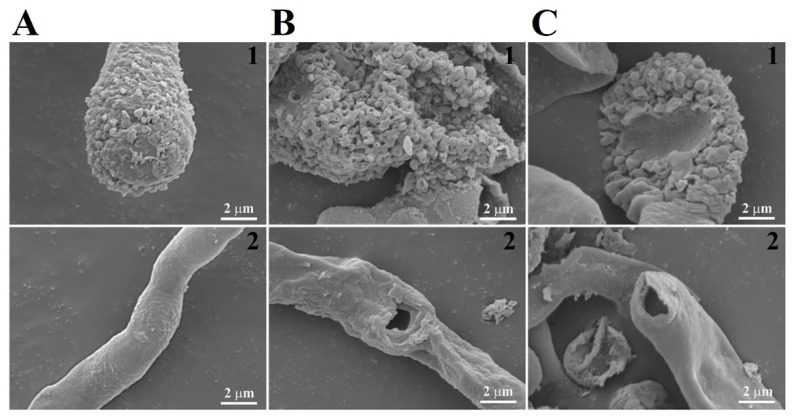
Morphological changes in CP4-EPSPS-treated *C. gloeosporioides* cells. Samples were incubated for 6 h at MICs, and cells were fixed, dehydrated, coated, and observed using a scanning electron microscope (SEM): (**A**), control; (**B**), CP4-EPSPS; (**C**), melittin; 1, Conidia; 2, hyphal.

Morphological changes in *C. gloeosporioides* were examined using SEM in the presence of melittin and CP4-EPSPS protein. After incubation for 6 h, melittin-treated cells showed wrinkled surfaces and irregular-sized holes on the cell surface (Figure 7C) compared to the typical morphology of untreated cells (Figure 7A). Cells treated with CP4-EPSPS at MICs had severely wrinkled cell surfaces and flattened cell morphology (Figure 7B), suggesting that the morphological changes were caused by CP4-EPSPS protein-induced physical damage. 

The major antimicrobial action of AMPs is to disrupt cell membranes through pore formation and permeability, resulting in rapid death and prevention of resistance emergence. Because their antimicrobial mechanism is different from conventional antibiotics used in animal agriculture and human medicine, the potential of AMPs as a new class of antibiotic has been suggested by many researchers. Antifungal proteins that induce cell surface permeability, thus, could have several applications. Other studies reveal that a vegetative insecticidal protein (Vip3Aa), another well-known protein expressed in LMO crops, induced the death of *Candida albicans* cells via the generation of mitochondrial reactive oxygen species and binding to nucleic acids [41]. However, this study found that the CP4-EPSPS protein induces cell surface permeability and kills *C. gloeosporioides*, a mold cell. Although the mechanism of the proteins may differ according to the difference in the cell wall components of yeast and mold cells, no cells were observed to expand or swell in yeast treated with CP4-EPSPS protein. Therefore, the antifungal mechanism of the CP4-EPSPS protein may be similar in the two different types of fungi.

## 3. Materials and Methods

### 3.1. Materials

Propidium iodide (PI), 5/6-carboxy-tetramethyl-rhodamine succinimidyl ester (NHS-Rhodamine), mitochondrial superoxide (MitoSOX) Red, and SYTOX Green were obtained from Thermo Fisher Scientific Inc. (Waltham, MA, USA). 9-Fluorenylmethoxycarbonyl (Fmoc) amino acids, ethyl 2-cyano-2-(hydroxyimino)acetate (Oxyma), and Rink Amide Protide resin (0.58 mmol/g) were purchased from CEM Co (Matthews, NC, USA). Trifluoroacetic acid (TFA) triisopropylsilane (Tis), and N,N’-diisopropylcarbodiimide (DIC) were obtained from Sigma-Aldrich Co. (St. Louis, MO, USA). All chemicals and solvents were more than 99.5% pure and were used exactly in the form received [40,41].

### 3.2. Fungal Strains 

*Candida albicans* (KCTC 7270), *C. tropicalis* (KCTC 7221), *C. krusei* (CCARM 14017), *Colletotrichum gloeosporioides* (KCTC 6169), *Fusarium solani* (KCTC 6326), *F. graminearum* (KCTC 16656), and *Trichoderma virens* (KCTC 16924) were obtained from the Korea Collection for Type Cultures (KCTC, Jeongup-si, Republic of Korea) and Culture Collection of Antimicrobial Resistant Microbes (CCARM, Seoul Women’s University, Seoul, Republic of Korea).

### 3.3. Cloning of CP4-EPSPS Gene and Expression of the Protein in Escherichia Coli

*CP4-EPSPS* gene was obtained by PCR amplification from an *Agrobacterium* sp. strain CP4. The gene was amplified using the following specific primers: CP4-EPSPS forward (5′-GGATCCATGGCGCAAGT-3′) and CP4-EPSPS reverse (5′-CTCGAGCGCCGCCTTGGTATCGCTC-3′). The gene was inserted into the overexpression vector pET28 (a) and transformed into *E. coli* cells. The transformant in BL21 (DE3) cells was cultured at 30 °C in a Luria–Bertani (LB) medium containing 50 μg/mL of kanamycin, and the CP4-EPSPS protein was induced using 0.5 mM isopropyl-β-D-thiogalacto-pyranoside (IPTG) at 22 °C. After harvesting the bacteria cells, the pellet was resuspended in binding buffer (50 mM sodium phosphate, 300 mM NaCl, 5 mM imidazole, and protease inhibitor, pH 8.0), after which the cells were lysed by sonication on the ice at 400 W for 10 min (sonication for 3 s and cooling for 3 s). Then, the supernatant separated by centrifugation at 10,000× *g* for 30 min at 4 °C was filtered using a 0.22-μm filter and applied to a Ni-NTA resin (Qiagen GmbH, Hilden, Germany). After washing to baseline absorbance with NTA wash buffer (50 mM sodium phosphate, 300 mM NaCl, and 30 mM imidazole, pH 8.0), bounded proteins were eluted by 70 or 250 mM imidazole in buffer (50 mM sodium phosphate and 300 mM NaCl, pH 8.0). The fractions were collected by analysis on 12% sodium dodecyl sulfate-polyacrylamide gel electrophoresis (SDS-PAGE), and the collected fractions were then dialyzed in phosphate-buffered saline (PBS, pH 7.2). The protein concentration was determined by Bradford’s method using bovine serum albumin, as a standard control. 

### 3.4. Peptide Synthesis

Melittin was prepared using solid-phase methods with Fmoc-protected amino acids and Rink Amide Protide resin on a Liberty Microwave Peptide synthesizer (CEM Co., Matthews, NC, USA). The coupling and deprotection processes were performed with Oxyma/DIC at 90 °C and 20% piperidine (*v*/*v*) in DMF at 110 °C heating, respectively. The final cleavage of peptides from the resin was performed by TFA/Tis/diH_2_O (95:2.5:2.5, *v*/*v*/*v*) solution for 40 min at 40 °C and the removed peptides were precipitated and washed with diethyl ether, followed by freeze-dry under lyophilizer. The crude peptides were isolated by a C18 column (Zorbax, 21.2 × 250 mm, 300 Å, 7-μm) on a Shimadzu semi-preparative HPLC system, using 20–80% acetonitrile gradient in water with 0.05% TFA for 60 min. The purity of the isolated melittin was measured on a HPLC system, and the molecular masses of the melittin were confirmed by using a matrix-assisted laser desorption ionization mass spectrometer (MALDI II, Kratos Analytical Ins, Manchester, UK). The purity of melittin used in this study was more than 95%.

### 3.5. SEC and PAGE

An Enrich SEC 650 column (Bio-Rad, Hercules, CA, USA) was used to purify the CP4-EPSPS protein. Using fast protein liquid chromatography (FPLC) (BioLogic DuoFlow Medium-Pressure Chromatography Systems, Bio-Rad), the column was equilibrated at 25 °C with PBS (pH 7.2) at a flow rate of 0.5 mL/min. After SEC, the protein was concentrated using a Centricon YM-10 unit (10 kDa cut-off, EMD Millipore, Billerica, MA, USA). The protein standards used for calibrating the SEC column included thyroglobulin (670 kDa), globulin (158 kDa), ovalbumin (44 kDa), myoglobin (17 kDa), and vitamin B12 (1.35 kDa) in the Gel Filtration Standard (Bio-Rad, Hercules, CA, USA) [40,42]. SDS-PAGE and native-PAGE analyses were performed as previously described [40,43].

### 3.6. Antifungal Assay

Yeast cells were cultured overnight in yeast extract–peptone–dextrose (YPD, Difco, Sparks, MD, USA) medium, and mold spores were collected with 0.08% Triton X-100 from 4-day-old cultures grown on potato dextrose (PD) (Difco) agar plates. Fungal suspensions in PBS (pH 7.2) containing 20% YPD for the yeasts or 20% PD for the molds (2 × 10^4^ spores (cells)/mL) were added to serially diluted CP4-EPSPS proteins and melittin peptides in 96-well plates (1000~15.6 µg/mL). The cell/mycelial growth of tested fungi was examined microscopically using an inverted light microscope after 24 h of incubation at 28 °C [40,44,45]. The minimum inhibitory concentration (MIC) of the protein or peptide against each fungus was determined as the lowest concentration that completely suppressed observable growth [40]. All assays were performed in triplicate.

### 3.7. Analysis Using Confocal Laser Scanning Microscopy (CLSM)

To label proteins with a fluorescent dye, rhodamine-NHS solution was added to CP4-EPSPS or melittin in PBS (pH 7.2) at a molar ratio of 1:1. After 2 h incubation with gentle agitation, the CP4-EPSPS/rhodamine mixture was dialyzed with PBS for 48 h or melittin/rhodamine mixture was applied on C_18_ RP-HPLC to remove the unreacted fluorescent dye. After incubating the rhodamine-labeled CP4-EPSPS or melittin samples with *C. gloeosporioides* cells at 28 °C for 2 h, the fungal cells were washed thrice with PBS using centrifugation (3000× *g*). The washed cells were mounted on a cover glass with a solution (50% glycerol and 0.1% n-propyl gallate) and examined under a CLSM (A1R HD 25, Nikon, Japan) [40,46].

### 3.8. PI uptake Assay

*C. gloeosporioides* conidial cells suspended in PBS (pH 7.2) with 10% YPD media at a density of 1 × 10^5^ conidia/mL were incubated with MICs of CP4-EPSPS or melittin for 2 h at 28 °C, and PI dye (10 µg/mL) was added to the cells. After 5 min, the fungal cells were investigated using a flow cytometer (Attune NxT acoustic focusing cytometer; Thermo Fisher Scientific Co.) [41].

### 3.9. SYTOX Green Uptake Assay

Preincubated *C. gloeosporioides* conidial cells were incubated with MICs of CP4-EPSPS or melittin for 2 h. The cells were incubated with SYTOX Green at 0.5 μM for 5 min in the dark, followed by analysis using fluorescence microscopy and flow cytometry (Attune NxT acoustic focusing cytometer) [42,47].

### 3.10. Mitochondrial Superoxide Assay

Reactive oxygen species (ROS) induction in *C. gloeosporioides* cells was measured using the MitoSOX Red probe. After incubating CP4-EPSPS or melittin with *C. gloeosporioides* for 4 h, fluorescent staining of cells was fulfilled following the manufacturer’s protocol for MitoSOX Red. The cells were investigated under a fluorescence microscope [41].

### 3.11. Scanning Electron Microscopy

MICs of CP4-EPSPS or melittin were incubated with *C. gloeosporioides* conidial cells (5 × 10^5^ conidia/mL) for 6 h, and the cells were fixed with 2% glutaraldehyde (*v*/*v*) in 0.2 M HEPES buffer (pH 8.0) overnight at 4 °C. The PBS-washed cells were postfixed in 1% osmium tetroxide (*w*/*v*) (Electron Microscopy Sciences, Hatfield, PA, USA) for 1 h and dehydrated using OTTIX Shaper (Diapath S.p.A, Bergamo, Italy), followed by chemical-drying using hexamethyldisilazane. The cells were sputter-coated with gold-palladium and observed using a scanning electron microscope (SEM) (JSM-7100F; JEOL, Ltd., Tokyo, Japan) [40,47].

## 4. Conclusions

In conclusion, this study provides additional knowledge about *Agrobacterium* CP4-EPSPS protein, which shows a broad spectrum of antifungal activity against seven fungi. It inhibited fungal growth through direct membrane damage after translocation into the cytosol through membrane integrity alteration and could also trigger cell death. However, many antifungal proteins, such as Vip3Aa and histatin5, induce apoptosis in fungal cells by elevating mitochondrial ROS generation [40,41]. The CP4-EPSPS protein may play a critical role in the *Agrobacterium* defense system against fungal pathogens and has the potential as an antifungal molecule. However, further investigation is required to determine its effectiveness for agricultural and clinical applications. Furthermore, if effective antifungal parts and domains can be defined in the CP4-EPSPS amino acid sequence, it will be possible to propose a new type of antifungal peptide.

## Figures and Tables

**Table 1:** Antifungal activity of CP4-EPSPS protein against fungal strains.

Fungal Strains	MIC (μg/mL) ^a^	
	CP4-EPSPS ^b^	Melittin
**Yeast**		
*C. albicans*	125	62.5
*C. tropicalis*	250	125
*C. krusei*	62.5	62.5
**Mold**		
*C. gloeosporioides*	62.5	500
*F. solani*	62.5	250
*F. graminearum*	62.5	500
*T. virens*	62.5	125

^a^ MIC, minimum inhibitory concentration; ^b^ CP4-EPSPS, 5-enolpyruvylshikimate-3-phosphate synthase (EPSPS) from *Agrobacterium* sp. strain CP4.

## Data Availability

Not applicable.

## Sample Availability

Not available.
